# Chronobiology discrepancies between patients with acute type a aortic dissection complicated with and without sleep apnea syndrome: a single-center seven-year retrospective study

**DOI:** 10.1186/s12872-023-03548-6

**Published:** 2023-10-12

**Authors:** Zeng-Rong Luo, Bin-Yao, Zhong-Yao Huang

**Affiliations:** 1 Department of Cardiovascular Surgery, Fujian Medical University Union Hospital, Fuzhou, 350001 P. R. China; 2 Key Laboratory of Cardio-Thoracic Surgery, Fujian Medical University, Fujian Province University, Fuzhou, P. R. China

**Keywords:** Chronological, Aortic dissection, Sleep apnea syndrome

## Abstract

**Background:**

The present study aimed to investigate the differences in chronobiology and prevention between patients with acute type-A aortic dissection (ATAAD) complicated with sleep apnea syndrome (SAS) and without sleep apnea syndrome (non-SAS).

**Methods:**

We retrospectively analyzed the clinical information of ATAAD patients using hospital medical records and regional meteorological and chronological information between January 2013 and December 2019.

**Results:**

An early mortality rate of 16.9% (196 out of 1160 cases) was observed, comprising 95 cases of aortic rupture before surgery and 101 surgery-related deaths. Eighty-one of the 964 survivors were screened for SAS using complete morphological characteristics. Of these patients, 291 (33.0%) suffered from SAS, while 590 (67.0%) had no SAS. Based on a Circular Von Mises distribution analysis, the non-SAS patients experienced a significant morning peak in the occurrence of ATAAD at 10:04 (r_1_ = 0.148, p < 0.01). In contrast, the SAS patients experienced a significantly different (non-SAS vs. SAS, *U*^2^ = 0.947, p < 0.001) nighttime peak at 23:48 (r_2_ = 0.489, p < 0.01). Moreover, both non-SAS (Z = 39.770, P < 0.001) and SAS (Z = 55.663, P < 0.001) patients showed a comparable peak during January (non-SAS vs. SAS, *U*^2^ = 0.173, p > 0.05). Furthermore, SAS patients experienced a peak on Fridays (χ^2^ = 36.419, p < 0.001), whereas there was no significant difference in the weekly distribution in non-SAS patients (χ^2^ = 11.315, p = 0.079).

**Conclusions:**

The analyses showed that both SAS and non-SAS patients showed distinct rhythmicity in ATAAD onset. These findings highlight the chronobiological triggers within different ATAAD subpopulations and may contribute to the prevention of this potentially fatal occurrence.

## Introduction

Acute type-A aortic dissection (ATAAD) is a serious disease with high rates of morbidity and mortality [1]. While significant progress has been made in the management of ATAAD, early detection of the condition remains a challenge. Epidemiological analysis has shown that ATAAD can occur at any time and may be induced by various factors [2]. Given the lack of a clear ATAAD etiology, understanding the underlying mechanisms may aid in preventing and detecting this disease.

Sleep apnea syndrome (SAS) is defined as the occurrence of ≥ 30 apnea events wherein the airflow ceases for a minimum of 10 s during a 7-hour single-night sleep, or ≥ 5 events of apnea per hour of sleep (apnea index ≥ 5) [3]. Recent research has found a relationship between SAS and cardiovascular disorders such as myocardial infarction, arrhythmias, and cerebrovascular disease (CVD) [4]. Further, SAS has been identified as a significant risk factor for the development of ATAAD [5], and SAS patients have shown a relatively high incidence of ATAAD [6].

Many reports have suggested the existence of specific chronobiological patterns in the onset of acute cardiovascular diseases, such as Takotsubo syndrome peaking in the afternoon [7] and myocardial infarction peaking in the morning [8]. Other studies have demonstrated a possible relationship between ATAAD onset and times of the day, week, month, and year [9–11]. However, few studies have investigated patterns of ATAAD onset in patients with SAS. It is not known whether patients with SAS exhibit typical or unusual patterns in ATAAD onset, such as seasonal, monthly, weekly, or chronobiological rhythms. We examined the temporal trends of ATAAD onset in SAS patients in Southeast China using a 7-year time-series analysis to answer this question. We believe our findings will be extremely useful in predicting and possibly preventing catastrophic implications for this patient population.

## Materials and methods

### Patient and public involvement statement

All patients provided written informed consent before using the portable sleep monitoring system (PSMS). Additionally, before extracting data from the electronic database, all personal patient data, including names and hospitalization numbers were removed to avoid a breach of patient confidentiality during our research.

### Participants and data acquisition

The patient data were from our hospital’s electronic medical database. This is a major hospital in southeast China and most patients are from the Chinese province of Fujian. The time-series data were obtained from ATAAD patients hospitalized between January 1, 2013, and December 31, 2019. To confirm the ATAAD diagnosis before inclusion in this study, the CT angiographical and transthoracic echocardiological data and the surgical records of the patients were carefully examined. Patients having computed tomographic angiogram (CTA) imaging or surgical records supporting the ATAAD diagnosis satisfied the inclusion criteria. The diagnosis of type-A aortic dissection (TAAD) was conclusive if an intimal tear was present in the ascending aorta, resulting in the formation of a false lumen within the aorta [12], identified from careful analysis of the CTA results by at least two experienced imaging specialists. ATAAD was defined if the time from onset to visit was less than two weeks. The following exclusion criteria were applied to investigate the patterns of ATAAD onset in terms of time distribution: 1, congenital aortic malformation; 2, Marfan syndrome; 3, connective tissue disease and vasculitis; 4, recent major organ surgery and trauma; 5, traumatic AAD/AAD during pregnancy. The date and time of ATAAD onset were determined from the documented onset of acute symptoms, as specified in the patient records. In this context, “typical symptoms” were defined if an acute appearance of intense chest or back pain.

**Fig. 1:**
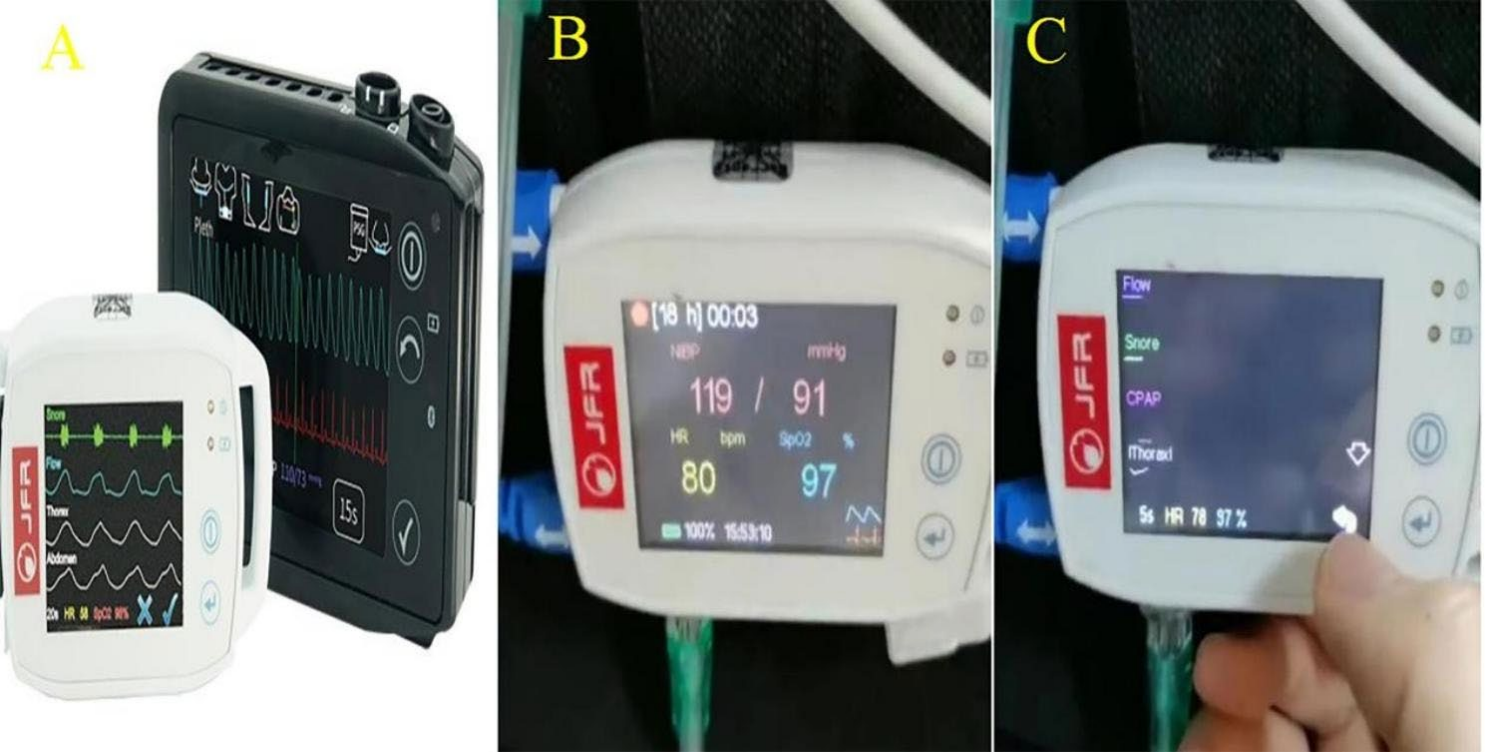
The PSMS which records relevant parameters **(A, B, C)** of individuals with SAS produced by JFR (Beijing) Digital Technology Co., Ltd. (PSMS, portable sleep monitoring system; SAS, sleep apnea syndrome.)

All surviving patients were asked to visit a certified regional outpatient clinic for a polysomnographic SAS examination approximately two weeks after discharge. SAS was diagnosed after the assessment of daytime drowsiness using the Epworth Sleepiness Scale (ESS) [13]. Generally, polysomnography is considered the optimal examination for a definitive diagnosis of SAS In this study, the patient’s sleep status was assessed over two consecutive nights using a portable sleep monitoring system (PSMS) [14], providing similar results to those obtained from polysomnography [14, 15]. The PSMS is a portable device designed for the regular recording and analysis of parameters related to SAS. It was developed by JFR Digital Technology Co., Ltd., Beijing (China), with a focus on managing breathing during sleep (Fig. 1) [16]. One senior electrocardiologist and one senior neurologist examined the sleep apnea data. Apnea was described as a blockage of oronasal airflow lasting for more than 10 s. The apnea index, computed by PSMS, is the average number of apnea episodes per hour of sleep. A patient was confirmed to have SAS if the apnea index was ≥ 5. Alternatively, a patient would be classified as “without SAS” (we have explicitly defined this designation in a clinical context as non-SAS). Respiratory inductance plethysmography was employed for recording the thoracoabdominal motion. SAS was classified as either obstructive sleep apnea (OSA) or central sleep apnea (CSA) based on the presence or absence of apparent thoracoabdominal breathing motion. Lastly, the severity of sleep apnea was classified using the stratification of the American Academy of Sleep Medicine Task: mild apnea index ≥ 5 to < 15; moderate, ≥ 15 to < 30; and severe, ≥ 30 [17].

### Study design

This study examined four temporal patterns of ATAAD onset, specifically, seasonal, monthly, weekly, and circadian patterns in both the non-SAS and SAS patient populations. Patients included in the study were analyzed retrospectively sequentially and who had died were eliminated from the analysis. The software PASS 15.0 can calculate the power of a retrospective study with a predetermined sample size. Therefore, we implemented the power calculation using PASS15.0 and obtained a statistical power value of 1.000. This suggested that the sample size in this retrospective study was adequate. The included patients were classified into four seasonal, 12 one-month, and seven one-day periods. The four seasons represented the regional climate and were classified as spring (March-May), summer (June-August), autumn (September-November), and winter (December-February). Circadian rhythmicity was also assessed in cases where the precise onset time was available. Lastly, the chronobiological patterns, particularly circadian variations, were compared between the SAS and non-SAS patients.

### Statistical analysis

Continuous data with normal distributions are presented as mean ± SD. Non-normally distributed data are expressed as median (first quartile [Q1]; third quartile [Q3]). Categorical data are presented as percentages. The uniformity of seasonal and weekly ATAAD onset distributions was assessed using the chi-square test. The circular distribution technique was applied for detecting relevant patterns and rhythmicity in monthly and circadian ATAAD onset, as detailed below.

The peak incidence was the average angle (α) when a concentration trend was evident. This value was converted from an onset time to an angle. For example, in the case of monthly data, 12 months (365 d) in 1 year became 360° and 0.9863° per day. The monthly median was the same as the group median and converted to angle αi. Therefore, the January median became 15.5°, February 45.5°, and so on. Likewise, in the case of circadian data, 24 h in 1 d became 360° and 15° per hour. The hourly median was the same as the group median and was converted to angle αi. Therefore, the first-hour median was 7.5°, the second-hour 22.5°, the third-hour 37.0°, and so on. The alpha of the Circular Von Mises distribution was used to reverse the prevalence peak duration. The validity of the peak distribution was confirmed further by the homogeneity test, which determined whether alpha was significant. Using the two-sample non-parametric *U* [2] test, we also compared the mean angle difference between the two patient populations. p < 0.05 was set as the significance standard. The Circular Von Mises distribution data were evaluated using the data processing system. The remaining statistical analyses were conducted using SPSS 26.0 statistical software.

## Results

### Subject demographics

**Fig. 2:**
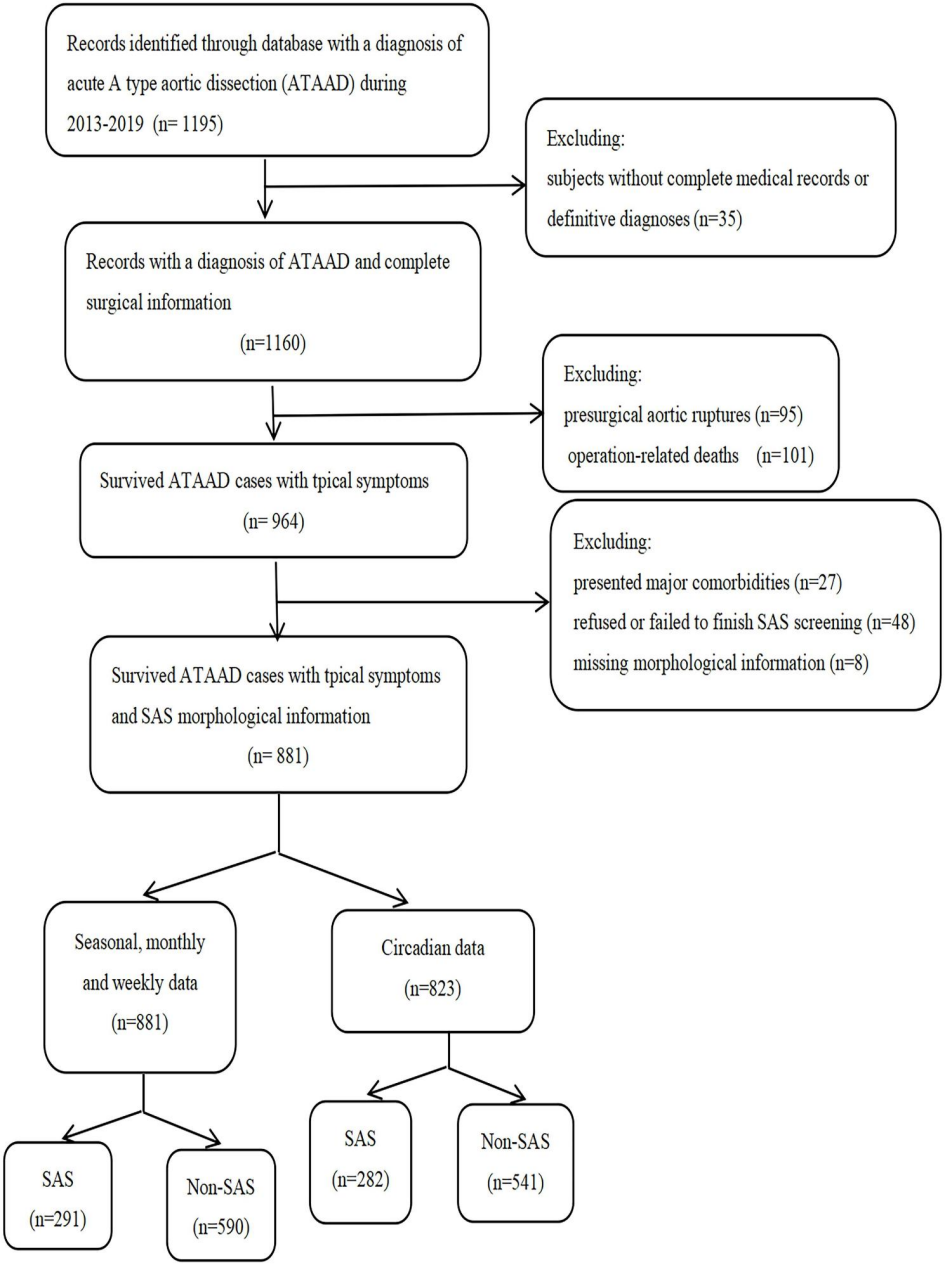
Flow chart of the methodology of the study

Among a total of 1195 patients, 1160 were identified as ATAAD patients with typical symptoms and definitive diagnoses. Of these, 16.9% (196 of 1160) experienced early mortality, including 95 cases of presurgical aortic ruptures and 101 in-hospital deaths. The patients with early mortality were eliminated from the final analysis. Among the 964 initially selected patients, 83 were excluded from analysis, including 27 patients with major comorbidities (10 cases of paralysis following acute stroke, 3 cases of metastatic cancer, 14 cases on dialysis), 48 patients who refused or failed to finish the SAS screening, and 8 patients who had missing morphological information. The final count of study participants was 881 after the exclusion of the aforementioned patients (Fig. 2).

The mean age of the 881 subjects was 56.4 ± 12.7 years, and 669 (75.9%) were male. In addition, 291 (33.0%) had SAS, while 590 (67.0%) did not. Approximately 79.7% and 16.7% of patients also had comorbid hypertension and diabetes, respectively. The two SAS and non-SAS groups showed similar proportions of hypertension (p = 0.362), nature of occupation (p = 0.522), and working time quantum (p = 0.265). Of 291 SAS ATAAD patients, 192 (66.0%) presented with obstructive sleep apnea (OSA) and 99 patients (34.0%) with central sleep apnea (CSA). Moreover, SAS patients had higher BMIs than non-SAS patients. Furthermore, it was observed that the overall location of aortic dissection intimal ruptures was similar between the two groups, and that the intimal rupture in patients with SAS appeared more likely to occur in the ascending aorta. Table 1 shows the demographic features of the patients.

**Table 1:** The demographics of 881 ATAAD patients

Variable	All patients	SAS	Non-SAS	t/χ^2^/Z	P value
	(N = 881)	(N = 291)	(N = 590)		
Age, year	56.9 ± 12.7	57.8 ± 12.9	56.5 ± 11.3	1.464	0.144
Male, n (%)	669 (75.9)	229 (78.7)	440 (74.6)	1.808	0.179
BMI	24.9 [22.9,26.4]	26.2[24.8,28.6]	23.2[21.8,25.6]	7.166	0.004
Hypertension ^a^, n (%)	702(79.7)	237 (81.4)	465 (78.8)	0.832	0.362
Diabetes ^b^, n (%)	147 (16.7)	50(17.2)	97(16.4)	0.077	0.781
Hypertriglyceridemia ^c^, n (%)	282 (32.0)	94 (32.3)	188 (31.9)	0.017	0.896
Acute kidney insufficiency ^d^, n (%)	222(25.2)	78 (26.8)	144 (24.4)	0.594	0.441
Malperfusion syndromes ^e^, n (%)	153 (17.4)	52 (17.5)	101 (17.3)	0.077	0.782
Systolic BP on arrival (mm Hg)	150.4 ± 29.7	152.6 ± 31.2	149.5 ± 30.6	1.405	0.160
Diastolic BP on arrival (mm Hg)	82.8 ± 20.0	83.2 ± 19.8	82.5 ± 21.5	0.466	0.641
**Ambulatory blood pressure monitoring**					
Non-Dipper, n (%)	407 (46.2)	169 (58.1)	238 (40.3)	24.665	< 0.001
Morning-night blood pressure difference (mm Hg)					
SBP difference	11.8 ± 3.9	10.3 ± 3.8	13.6 ± 3.9	11.912	< 0.001
DBP difference	10.5 ± 3.8	8.4 ± 3.6	11.0 ± 4.0	9.713	< 0.001
Nature of occupation, n (%)				0.411	0.522
Manual worker	314 (35.6)	108 (37.1)	206 (34.9)		
Non-manual worker ^f^	567 (64.4)	183 (62.9)	384 (65.1)		
Working time quantum, n (%)				1.245	0.265
Night shift	210 (23.8)	76 (26.1)	134 (22.7)		
Day shift	671 (76.2)	215 (73.9)	456 (77.3)		
Location of the aortic dissection intimal rupture, n (%)				4.989	0.083
Ascending aorta	642 (72.9)	225_a_ (77.3)	417_b_ (70.7)		
Aortic arch	183 (20.8)	53_a_ (18.2)	130_a_ (22.0)		
Descending aorta	56 (6.4)	13_a_ (4.5)	43_a_ (7.3)		

^a^ Defined as systolic blood pressure higher than 140 mmHg and (or) diastolic blood pressure higher than 90mmHg

^b^ Defined as variable venous plasma glucose: occasional plasma glucose value of ≥ 200 mg/dl, or fasting plasma glucose of ≥ 126 mg/dl, or OGTT 2-h value in venous plasma ≥ 200 mg/dl

^c^ Defined as fasting serum triglycerides greater than150 mg/dl

^d^ Defined as serum creatinine greater than 2.0 mg/dl according to Society of Thoracic Surgery

^e^ Defined as compromised blood flow in one or more organs resulting in ischemia and organ dysfunction

^f^ Defined as the occupational groups of the first and second categories according to the Occupational Classification Code of the People’s Republic of China

Data are expressed as mean ± standard deviations (SD), median [first quartile (Q1); third quartile (Q3)] or number (%). SAS, sleep apnea syndrome

“_aa_” represents there is no significant difference between the column proportions of these categories (by Bonferroni method);

“_ab_” represents there is significant difference between the column proportions of these categories (by Bonferroni method);

Non-SAS, without sleep apnea syndrome. BMI, body mass index. BP, blood pressure

### Seasonal and weekly patterns

**Fig. 3:**
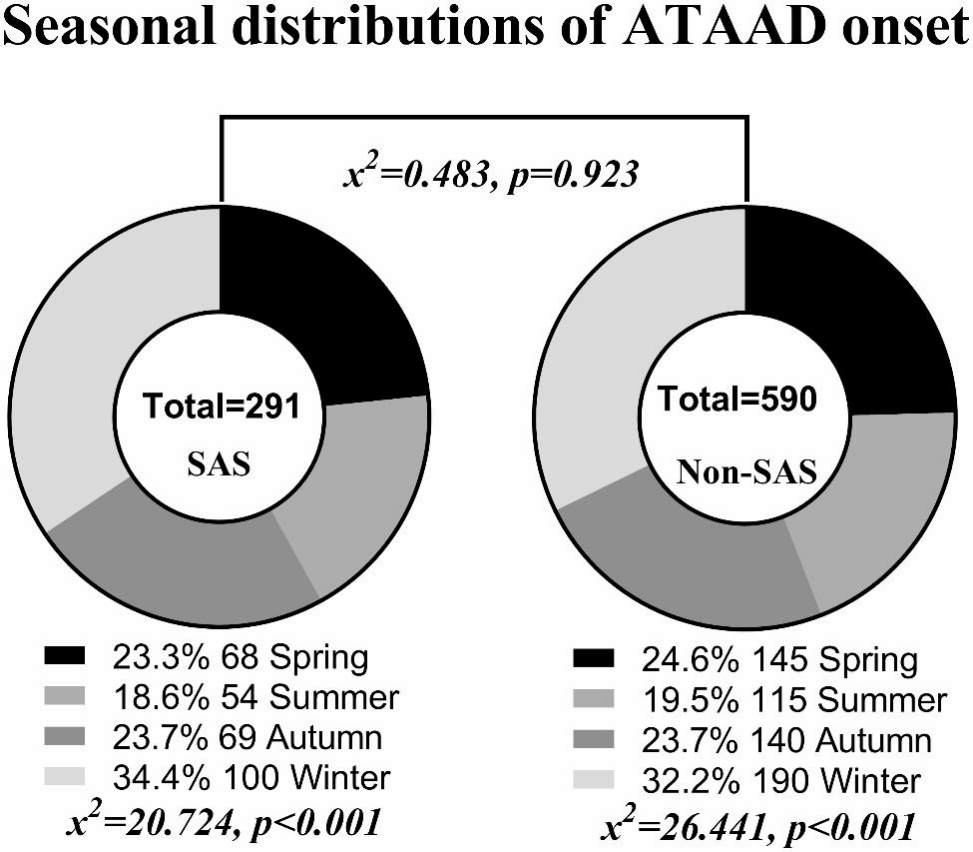
Seasonal patterns of ATAAD onset in the SAS and non-SAS groups. (ATAAD, acute aortic dissection; SAS, sleep apnea syndrome; Non-SAS, without sleep apnea syndrome.)

One hundred ATAAD onsets (34.4%) were seen in SAS patients during the winter and 54 (18.6%) in the summer (χ^2^ = 20.724, p < 0.001). Non-SAS patients, similarly, mainly had winter ATAAD onsets (32.2%, 190 cases), with fewer onsets in the summer (19.5%, 115 cases) (χ^2^ = 26.441, p < 0.001). However, the seasonal ATAAD distribution between the two patient populations was not statistically significant (χ^2^ = 0.483, p = 0.923) (Fig. 3).

**Fig. 4:**
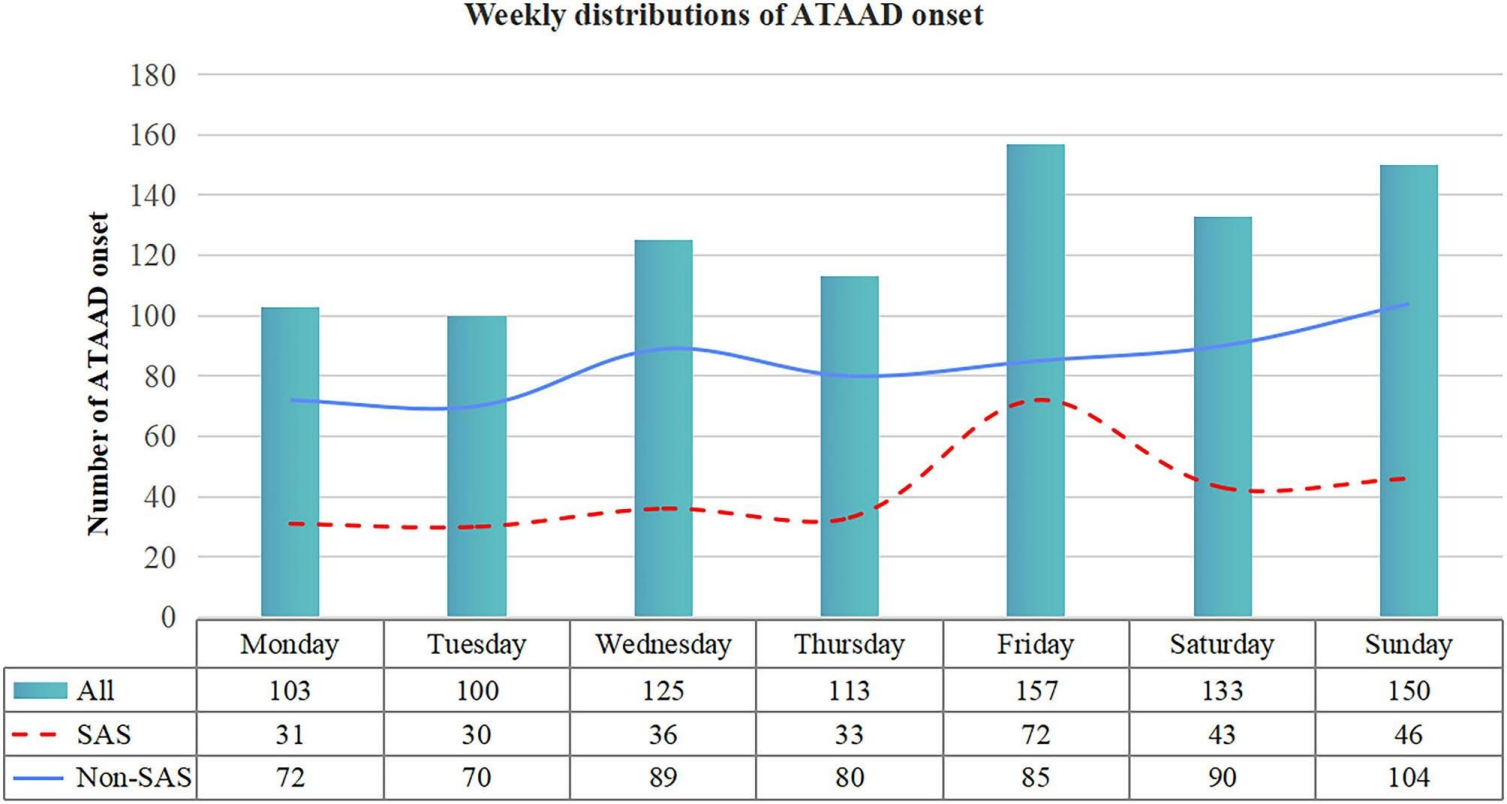
Weekly patterns of ATAAD onset in the SAS and non-SAS groups. (ATAAD, acute aortic dissection; SAS, sleep apnea syndrome; Non-SAS, without sleep apnea syndrome.)

Figure 4 illustrates the weekly ATAAD distribution. Interestingly, this differed significantly between the non-SAS and SAS patient populations (χ^2^ = 14.666, p = 0.023). A peak was observed on Fridays (χ^2^ = 36.419, p < 0.001) in the SAS subgroup, while no apparent weekly distribution was seen in the non-SAS patients (χ^2^ = 11.315, p = 0.079).

### Monthly and circadian patterns

Using the circular distribution method, a peak was observed in January (r_2_ = 0.296, α = 15.933 °, P < 0.01) with 15.71 converted days (i.e., January 15 to 16) in SAS patients. Similar patterns were also observed in the non-SAS patients (r_1_ = 0.168, α = 10.506 °, P < 0.01) with 10.36 converted days (i.e., January 10 to 11). The homogeneity test further confirmed a significantly concentrated ATAAD pattern in both SAS (Z = 55.663, P < 0.001) and non-SAS patients (Z = 39.770, P < 0.001). However, there was no significant difference in the monthly distribution of ATAAD between the two patient cohorts (non-SAS vs. SAS, *U*^2^ = 0.173, p > 0.05) (Table 2).

**Table 2:** Monthly variations of ATAAD onsets, fitted by circular Von Mises distribution and non parametric U2-test

Group	N	α	r	Monthly peak	Z	P value	*U* [2] (Non-SAS vs. SAS)	P (Non-SAS vs. SAS)
Non-SAS	590	α1 = 10.506 °	r_1_ = 0.168	January peak	39.770	< 0.001	0.173	> 0.05
SAS	291	α2 = 15.933 °	r_2_ = 0.296	January peak	55.663	< 0.001		

SAS, sleep apnea syndrome; Non-SAS, without sleep apnea syndrome

Of the 881 patients, 823 had documented ATAAD onset times in their medical records (282 with SAS and 541 without). These patients were entered into the circadian analysis. The Circular Von Mises distribution analysis detected a significant morning peak at 10:04 in the non-SAS patient population (r_1_ = 0.148, p < 0.01) and a significant night peak at 23:48 in the SAS patient population (r_2_ = 0.489, p < 0.01). The two-sample non-parametric *U* [2]-test identified a different circadian pattern between the non-SAS and SAS patient populations (*U*^2^ = 0.947, p < 0.001) (Table 3).

**Table 3:** Circadian variations of ATAAD onsets, fitted by circular Von Mises distribution and non parametric U2-test

Group	N	r	Morning peak	r	Night peak	P value	*U* [2] (Non-SASvs.SAS)	P (Non-SAS vs. SAS)
Non-SAS	541	r_1_ = 0.148	10:04			< 0.01	0.947	< 0.001
SAS	282			r_2_ = 0.489	23:48	< 0.01		

SAS, sleep apnea syndrome; Non-SAS, without sleep apnea syndrome

## Discussion

In this study, a single-center retrospective investigation was conducted, involving 881 eligible surviving ATAAD patients from Southeast China, to explore potential chronobiological trends in ATAAD onset in both SAS and non-SAS patient populations.

First, ATAAD onset in both SAS and non-SAS patients was observed to peak in the cold month of January, followed by a decline in the warm month of July. This finding is consistent with those previous investigations [9, 18]. Reduced temperature is usually correlated with augmented sympathetic activity, blood pressure (BP), and changes in hematological properties, including increased blood viscosity, hemoglobin, mean corpuscular volume, platelet aggregation, factor VII, fibrinogen, and acute-phase reactants, as well as diminished red cell deformability [2, 9]. In addition, air pollutants, mainly fine particulate matter, tend to increase during the winter season and have been associated with aortic plaque progression, hypertension, and aortic constriction [10]. These changes can significantly increase shear forces on the aortic intima, thereby inducing ATAAD in both SAS and non-SAS patients during winter.

Second, new findings on the weekly distribution of ATAAD onset are described. Several studies have reported a Monday peak [18, 19], whereas others have found a Wednesday peak [20, 21]. The Monday peak can be explained as likely due to emotional stress associated with the commencement of weekday work-related activities [22], less favorable biochemical factors such as carbohydrate and lipid contents [23], and elevated BP levels on Mondays [24], compared to the rest of the week. No noticeable variation in ATAAD onset was observed during the week in the non-SAS patient population. However, here, a Friday peak was observed in SAS patients, likely due to activation of the sympathetic nervous system (SNS) by anticipation of weekend over-indulgences, such as going to bed late, in addition to the greater susceptibility of SAS patients to ATAAD. According to earlier reports, SAS exacerbates the development of atherosclerotic disease by promoting the escalation and maintenance of SNS-mediated hypertensive conditions [25, 26] and chronically elevated mechanical stress on the aortic wall [6]. SAS likely involves a multifactorial pathway that accelerates the pathogenesis of ATAAD. Similarly, the weekly distribution of cardiovascular disease (CVD) may reflect the normal fluctuations of physical and emotional challenges associated with transitioning from a leisurely weekend to a stressful work week [27]. Therefore, fluctuations in employment status and seasonal holidays might influence the weekly distribution of ATAAD onset, thus resulting in a non-significant difference.

Regarding circadian variation, similar to reports documenting a morning peak in numerous adverse CVD-related incidents such as stroke, myocardial infarction, and sudden cardiac death [28], a significant morning peak (10:04) was observed in ATAAD onset in non-SAS patients. Over the last few decades, scientists have demonstrated that human BP readings follow a circadian pattern. This may be due to the interaction of the intrinsic circadian clock with external environmental perturbations and everyday actions. For example, BP is reduced with resting at night but rises rapidly the following morning, known as the ‘morning surge.‘ [11] Interestingly, most cardiovascular parameters display a circadian rhythm with a morning peak. This includes the heart rate, renin-angiotensin-aldosterone network activity, vascular resistance, and sympathetic activity [6]. Furthermore, the coagulation and fibrinolysis balance is also disrupted, resulting in hypercoagulability and hypofibrinolysis in the morning [14]. Elevated BP and BP reactivity and other changes in the cardiovascular system increase the arterial shear force, potentially contributing to the development of ATAAD, particularly in individuals with a genetic predisposition.

The chronobiological features of ATAAD onset in patients with SAS were further investigated. The circadian analyses were carried out in SAS patients to determine whether ATAAD onset follows a typical or unique circadian pattern, identifying certain significant differences. First, SAS patients experienced higher nocturnal BP (non-dipper) with smaller morning-night drops in systolic BP (P < 0.001) and diastolic BP (P < 0.001). In addition, these patients had a significantly higher proportion of non-dipper nocturnal BP patterns. This explains why SAS patients tended to experience an increase in nighttime ATAAD onset (23:40) relative to non-SAS patients (10:04) in our study. This could increase the impact of SAS on irregular circadian variations in autonomic balances. Internal and external mechanical stress caused by wandering excessive BP has been associated with the development of ATAAD. In the present study, together with the characteristic non-dipping or riser pattern of nocturnal BP profiles [29, 30], the SAS group showed higher readings during the night, increasing the amplitude and variability of BP during sleep, contributing to the incidence of ATAAD. Seguchi et al. also suggested that nocturnal hypertension in SAS could be a risk factor for ATAAD after evaluating circadian variations in BP in patients with spontaneous ATAAD using ambulatory BP monitoring despite controlled BP during the daytime [31]. This feature of uncontrolled nocturnal hypertension with a nocturnal BP surge was more noticeable in OSA patients on ambulatory BP monitoring [30]. Consequently, we found that there was a higher proportion of patients with obstructive sleep apnea (OSA) than central sleep apnea (CSA) among ATAAD patients.

Moreover, based on earlier studies, the involvement of SAS in ATAAD pathogenesis is associated with many other factors: (1) nocturnal negative intrathoracic pressure surges that mechanically stretch the aorta, thus causing distension; (2) nocturnal arousal-driven reflex sympathetic activation that worsens and persists hypertensive conditions [25, 26]; (3) nocturnal intermittent hypoxia (IH) and re-oxygenation correlated with the autonomic nervous system stimulation; (4) subsequent rises in oxidative stress [32–34]. The association between SAS and ATAAD may justify the higher nighttime rise in ATAAD in SAS patients. A bedtime dose of α-adrenergic blockers decreased the nighttime BP surge in OSA patients [31], demonstrating that sympathetic nervous activation and subsequent vasoconstriction are the leading causes of the midnight BP surge. This finding may also strengthen the effect of SAS on the irregular circadian variation in autonomic balance. Moreover, the mechanism by which OSA affects aortic dissection (AD) has been further investigated with the help of the newly established OSA-AD in vivo mouse model and in vitro cell culture studies, which demonstrates OSA-induced nocturnal IH can promote the occurrence and progression of AD via a ROS-HIF-1α-MMPs associated pathway [34]. However, further investigations are warranted to examine the role of SAS, ATAAD, and the associated chronobiological pattern in people of other ethnicities.

After the diagnosis of SAS, the patients in the present study were all started on Continuous Positive Airway Pressure (CPAP) treatment with a portable ventilator at night. As noninvasive respiratory support, CPAP is considered a “gold standard” for treating respiratory failure caused by SAS [35]. It helps to prevent upper-airway obstruction, thereby avoiding BP elevations due to negative intrathoracic pressure and sympathetic activation, thus improving nocturnal hypertension.

We investigated the seasonal, monthly, weekly, and particularly circadian ATAAD onset patterns in SAS patients over seven years, and the results revealed some noteworthy characteristics in patients from Southeastern China who suffered from ATAAD. Equipped with the knowledge of the ATAAD onset time rhythms in SAS patients, clinicians can recommend chronotherapy for this patient population. For example, keeping warm in winter, avoiding working at night at weekends, and staying up late during the transition from weekday to weekend can lower the occurrence of ATAAD in SAS patients. At the same time, using the nighttime anti-hypertensive medication can reduce BP fluctuations during the evening, thus reducing morbidity and mortality in these patients [26]. Recent developments in artificial intelligence (AI) systems could be beneficial in precision medicine to improve outcomes by the early identification of patient subgroups that may react negatively to specific treatments [36]. Finally, the majority of these patients will benefit from a timely initiation of noninvasive respiratory support and the effective treatment of arterial hypertension [37].

### Limitations

Our work encountered certain limitations. Firstly, the retrospective study only focused on surviving cases which may have introduced detection/diagnostic and selection bias. Memory bias may have also been introduced as the data were obtained from the electronic medical records. Furthermore, because the study was retrospective, it was diffcult to determine whether patients had dysregulated hypertension and variations in the sleep and waking cycles and hypertension medication might alter the innate circadian pattern of ATAAD onset. Finally, it is essential to note that the study was limited to a single center, raising concerns about its generalizability to the broader population. To address these challenges, conducting a study with a broader sample of patients from multiple centers is recommended. This approach would enable a more extensive investigation into the circadian patterns associated with the onset of ATAAD in patients with SAS.

## Conclusions

**Fig. 5:**
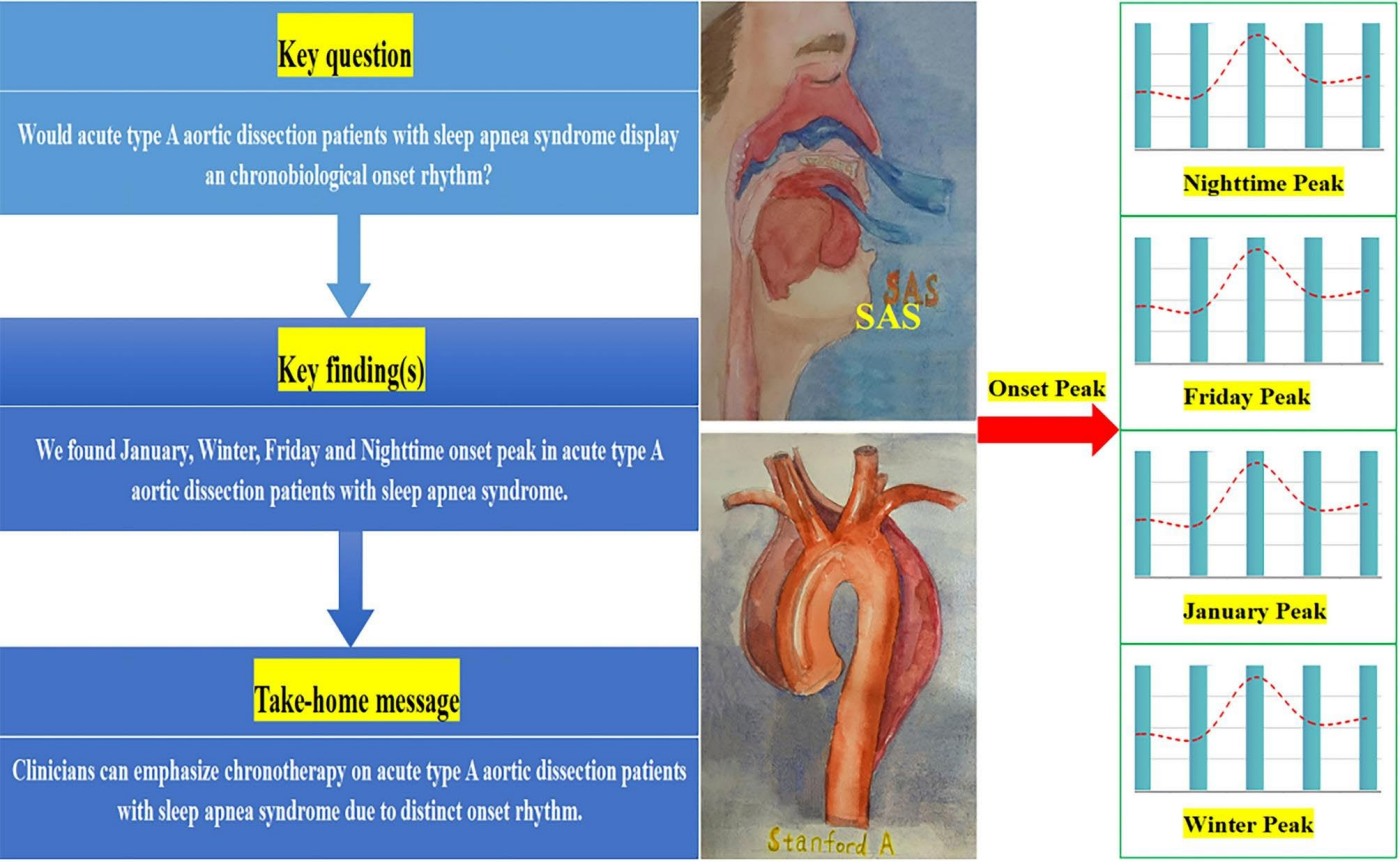
A retrospective summary of the findings of the current study

The findings demonstrated distinct seasonal, weekly, and circadian rhythm in a ATAAD population retrospectively screened for SAS. We observed an increase in ATAAD during the winter, specifically in January, in both the non-SAS and SAS patients. In terms of weekly and circadian fluctuations, there was a peak on Fridays and a nighttime-onset peak (23:40) in SAS patients (Fig. 5). Thus, paying more attention to circadian BP fluctuations and advocating for the use of chronotherapy as a recommended treatment approach for individuals with SAS are imperative.

## Data Availability

The data that support the findings of this study are available from Fujian Cardiac Medical Center but restrictions apply to the availability of these data, which were used under license for the current study, and so are not publicly available. Data are however available from the corresponding author upon reasonable request and with permission of Fujian Cardiac Medical Center.

## Abbreviations

ATAAD: Acute aortic dissection
BMI: Body mass index. BP, blood pressure
CPAP: Continuous positive airway pressure
CVD: Cardiovascular disease
IH: Intermittent hypoxia
Q1: First quartile
Q3: Third quartile
SAS: sleep apnea syndrome
